# Impact of different concentrations of biogas slurry as a fertilizer substitute on the structure and gene distribution of microbial communities in oilseed rape soil

**DOI:** 10.3389/fvets.2025.1563124

**Published:** 2025-03-28

**Authors:** Xue Yang, Kai Zhang, YaYing Chen, Cancai Zheng, Chunlong Lei, Xiaoqin Song, Bin Wen, Xin Yuan, Hanyang Liu, Jiawen Zhu

**Affiliations:** ^1^Animal Husbandry Research Institute, Chengdu Academy of Agricultural and Forestry Sciences, Chengdu, China; ^2^Institute of Herbivorous Livestock Research, Sichuan Academy of Grassland Sciences, Chengdu, Sichuan, China; ^3^Sichuan Provincial Planning and Construction Service Center, Chengdu, Sichuan, China; ^4^Dayi County Agriculture and Rural Bureau, Chengdu, Sichuan, China; ^5^Sichuan Agricultural University, Chengdu, Sichuan, China

**Keywords:** soil microorganisms, oilseed rape, sustainable agriculture, fertilizer replacement, antibiotic resistance genes

## Abstract

Application of biogas slurry (BS) in paddy fields offers a sustainable approach to reducing environmental risks while efficiently utilizing nutrients from biogas residues. However, BS may harbor animal gut microorganisms and associated antibiotic resistance genes (ARGs) arising from antibiotic use during livestock breeding, potentially facilitating the spread of resistance genes in soil ecosystems. In this two-year field study, we investigated the effects of replacing chemical fertilizers with different proportions of BS on the microbial communities in oilseed rape soils. Soil bacterial community diversity and composition were systematically evaluated using Illumina MiSeq high throughput sequencing, focusing on interrelationships and changes at the genetic level. The predominant bacterial phyla identified were *Proteobacteria, Actinobacteria*, and *Bacteroidetes*, with no significant differences observed at the phylum or species level across treatment groups. Overall microbial diversity and community structure remained largely stable. Dimensionality reduction analysis further revealed only minor variations in the abundance of ARGs, mobile genetic elements (MGEs), and metal resistance genes (MRGs) among treatments. These findings suggest that, in the short term, substituting chemical fertilizers with BS has minimal effects on soil microbial community structure and resistance gene dynamics. This study highlights the potential of BS as a viable alternative to chemical fertilizers, contributing to reduced environmental risks in agriculture. Future research should investigate the long-term ecological safety of BS under different soil types and management practices to comprehensively evaluate its sustainability in agricultural systems.

## 1 Introduction

With the rapid development of modern agriculture, traditional farming practices have relied heavily on large amounts of chemical fertilizers, pesticides, and herbicides to ensure crop yields. However, this overreliance on chemical inputs has caused significant environmental impacts, leading to a decline in soil microbial community diversity and causing pollution of water and soil resources (1, 2). In light of the current environmental pressures faced by agriculture, exploring more environmentally friendly and sustainable agricultural strategies has become particularly important. Biogas, as a clean energy source, has attracted widespread attention in recent years (3). Produced as a by-product of anaerobic digestion of organic waste, BS is rich in nutrients and low in cost, and has gradually been regarded as a promising alternative to chemical fertilizers (4, 5).

Biogas is primarily composed of methane (50%−70%) and carbon dioxide (30%–50%), as well as minor constituents like hydrogen sulfide and nitrogen. Additionally, BS—the liquid by-product remaining after anaerobic digestion—contains trace amounts of amino acids, nitrogen, phosphorus, potassium, and other micronutrients (6, 7). The substrates used for anaerobic digestion to produce biogas are highly diverse, typically including animal waste (e.g., livestock and poultry manure, feed residues), plant materials (e.g., forage grasses and crop straw), and domestic waste (e.g., kitchen scraps and manure). Among these, livestock manure, especially cattle manure, is the most commonly used and cost-effective substrate for biogas production due to its wide availability and abundant supply in rural areas (8, 9). BS has been reported to significantly affect soil nutrient status, crop growth, and soil health. Studies have shown that the appropriate application of BS can enhance crop nutritional quality, increase soil nutrient content, and promote plant growth and stress resistance (10–13). The study revealed that BS treatment significantly inhibits root exudation in Brassica napus, redirecting carbon and nitrogen resources toward reproductive organ development, thereby enhancing reproductive growth and achieving measurable yield improvement (14). Another study reveals that substituting 30% of synthetic fertilizers with organic amendments enhances nutrient use efficiency, increases yield, and improves quality metrics in cabbage cultivated under intensive open-field systems (15). Therefore, using BS directly as fertilizer not only significantly reduces the cost of hazardous waste treatment but also improves crop yields and reduces overall agricultural inputs (16). However, as a typical liquid organic waste, the impact of BS on soil microbial communities remains controversial. Some studies have indicated that BS application does not significantly affect bacterial α-diversity or microbial enzyme activity (17–19). In contrast, Xu et al. (20) reported that moderate doses of BS can improve bacterial diversity, but excessive doses may lead to negative effects, such as the accumulation of heavy metals, organic pollutants, and water pollution (13, 21). However, Considerable quantities of heavy metals are present in biogas slurry (22). Continuous irrigation with biogas slurry potentially induces the accumulation of heavy metals in both soil and crops (23, 24). Additionally, widespread studies indicate that BS promotes the dissemination of antibiotic resistance genes, subsequently giving rise to environmental and health issues (25, 26).

Soil is widely recognized as a reservoir of diverse microbial resources, containing approximately one billion microorganisms per gram of soil (27). These microbial communities play a critical role in maintaining ecosystem functions, including nutrient cycling, organic matter decomposition, and crop health (28–30). Consequently, shifts in the diversity and functionality of soil microbial communities are considered key indicators for evaluating soil health and agricultural sustainability (31, 32). Previous studies have shown that the application of BS significantly affects the structure and function of soil microbial communities. For instance, BS application has been shown to increase the relative abundance of methanogens and acetogens, thereby enhancing nutrient cycling efficiency in soils (33). However, its impact on fungal communities appears more limited and may even reduce fungal diversity in certain conditions (34). The effects of BS are not uniform but are modulated by factors such as soil type, application rate, and environmental conditions (35, 36). The vast majority of microorganisms in nature are difficult to isolate and culture using traditional methods, which greatly limits our in-depth understanding of microbial diversity and function (37–39).

In recent years, rapid advancements in high-throughput sequencing technologies have greatly enhanced our understanding of microbial communities (40, 41). These technologies enable researchers to optimize microbial community structures and explore strategies to enhance sustainable agriculture and regulate soil nutrient availability (42, 43). Building on these advancements, this study employs metagenomic analysis to investigate the compositional and functional changes in soil microbial communities when varying concentrations of BS replace chemical fertilizers in oilseed rape soils. By analyzing antibiotic resistance genes (ARGs), mobile genetic elements (MGEs), and metal resistance genes (MRGs), we hope to reveal the potential ecological functions of BS in soil microbial communities, provide a theoretical basis for its rational application, and promote sustainable agricultural development.

## 2 Materials and methods

### 2.1 Site description and experimental design

The experimental site was located at a farm in Dayi County, Sichuan Province, China (30°35′17″N, 103°31′23″E). This region is characterized by a subtropical humid monsoon climate with hot and rainy summers and mild, humid winters. Average annual temperatures range from 16°C to 18°C, with annual rainfall around 1,200–1,500 mm. The field adopted a rice-oilseed rape rotation system. The experiment was conducted over two seasons in each year: the early season (September 2021–May 2022 and September 2022–May 2023) and the late season (May 2022–September 2022 and May 2023–September 2023), concluding after oilseed rape harvest. Under a total fertilization rate of nitrogen:phosphorus at 180:90:180 kg/hm^2^, BS was used to partially replace chemical fertilizers at different gradients with BS/chemical fertilizer ratios of: no fertilizer (Blank), 0:100 (P0), 15:85 (P15), 30:70 (P30), 50:50 (P50), 75:25 (P75), and 100:0 (P100). Each treatment was replicated five times with each plot covering an area of 15 m^2^ (3 × 5 m). The total amount of fertilizer applied was consistent across all treatments, with the BS sourced from the farm's anaerobic fermentation tank for pig manure and stored in a sedimentation tank (44). The supernatant from the BS was applied to the paddy fields via cement irrigation channels, mixed with irrigation water. The total water volume applied was uniform across all plots. Before applying BS, we confirmed that there would be no heavy rainfall in the following 48 h.

### 2.2 Soil sampling and chemical property analysis

After the oilseed rape crop reached maturity, soil samples were collected from the 10–20 cm depth using a soil auger, targeting the root zone where microbial activity is most pronounced. Approximately 1.5 kg of wet soil was obtained from each plot. The collected soil was divided into two portions: one portion was air-dried under ambient laboratory conditions (22–25°C) with periodic mixing to ensure uniform drying, which was used to measure physicochemical indicators. The second portion was aseptically transferred into individual sterile cryovials, immediately flash-frozen in liquid nitrogen, and stored at −80°C to preserve microbial DNA integrity for subsequent community analysis. The physicochemical parameters measured included pH, moisture content, organic matter, total nitrogen, total phosphorus, total potassium, arsenic, mercury, lead, cadmium, chromium, copper, and zinc. These indicators were selected to assess both soil fertility and potential environmental risks associated with heavy metal accumulation.

### 2.3 Metagenomic sequencing

Sequencing was conducted on an Illumina NovaSeq 6000 platform (Illumina Inc., San Diego, CA, USA) at Majorbio Bio-Pharm Technology Co., Ltd. (Shanghai, China) using the NovaSeq 6000 S4 Reagent Kit v1.5 (300 cycles) according to the manufacturer's instructions. DNA extracts were fragmented into approximately 400 bp fragments using a Covaris M220 (Gene Company Limited, China). Paired-end libraries were prepared with the NEXTflex™ Rapid DNA-Seq kit (Bioo Scientific, Austin, TX, USA), where adapters containing all sequencing primer hybridization sites were ligated to the blunt ends of the fragments. The raw metagenomic reads have been deposited in the CNCB Sequence Read Archive database (Accession No.: PRJCA033921). We conducted quality control on the raw reads using Trimmomatic v0.39 (45) with the following parameters: –threads 30 LEADING:3 TRAILING:3 SLIDINGWINDOW:5:20 MINLEN:60, by removing adapter sequences and trimming low-quality reads. Host contamination was removed by mapping all trimmed reads to the wheat reference genome (GCF_034140825.1) using Bowtie2 v2.5.1 (46). Quality reports for the metagenomic reads of each sample were generated using Seqkit v2.6.1 (47). To reconstruct microbial genomes from the metagenomic data, we employed SPAdes v3.15.5 (48) for metagenome assembly and contig construction. Additionally, we merged contigs assembled from all samples and used Prodigal v2.6.3 (49) to construct a complete gene set, followed by redundancy removal using CD-HIT v4.8.1 (50).

### 2.4 Statistical analysis

All statistical analyses were performed using R software (version 4.2.0; https://www.r-project.org) (51), ensuring compatibility with the latest statistical packages. Alpha and beta diversity of microbial communities were analyzed using QIIME2 (version 2022.2) (52), where alpha diversity metrics (e.g., Shannon index, Chao1 richness) and beta diversity distances (e.g., Bray-Curtis dissimilarity) were calculated to evaluate community structure. Analysis of variance (ANOVA) and permutational multivariate analysis of variance (PERMANOVA) from the “vegan” R package were conducted to assess the effects of different BS concentrations on soil gene functions, including those related to nutrient cycling and heavy metal resistance. Linear discriminant analysis effect size (LEfSe) was used to identify key bacterial taxa and biomarkers significantly associated with BS treatments, using a linear discriminant analysis (LDA) effect size threshold of 2.0 to ensure robust differentiation (53). All analyses were conducted with a significance level set at *p* < 0.05, and the *p*-values were adjusted using the Benjamini-Hochberg FDR correction. The gene annotation screening threshold was uniformly set at 1e-5.

## 3 Results

### 3.1 Soil property characteristics

**Figure 1:**
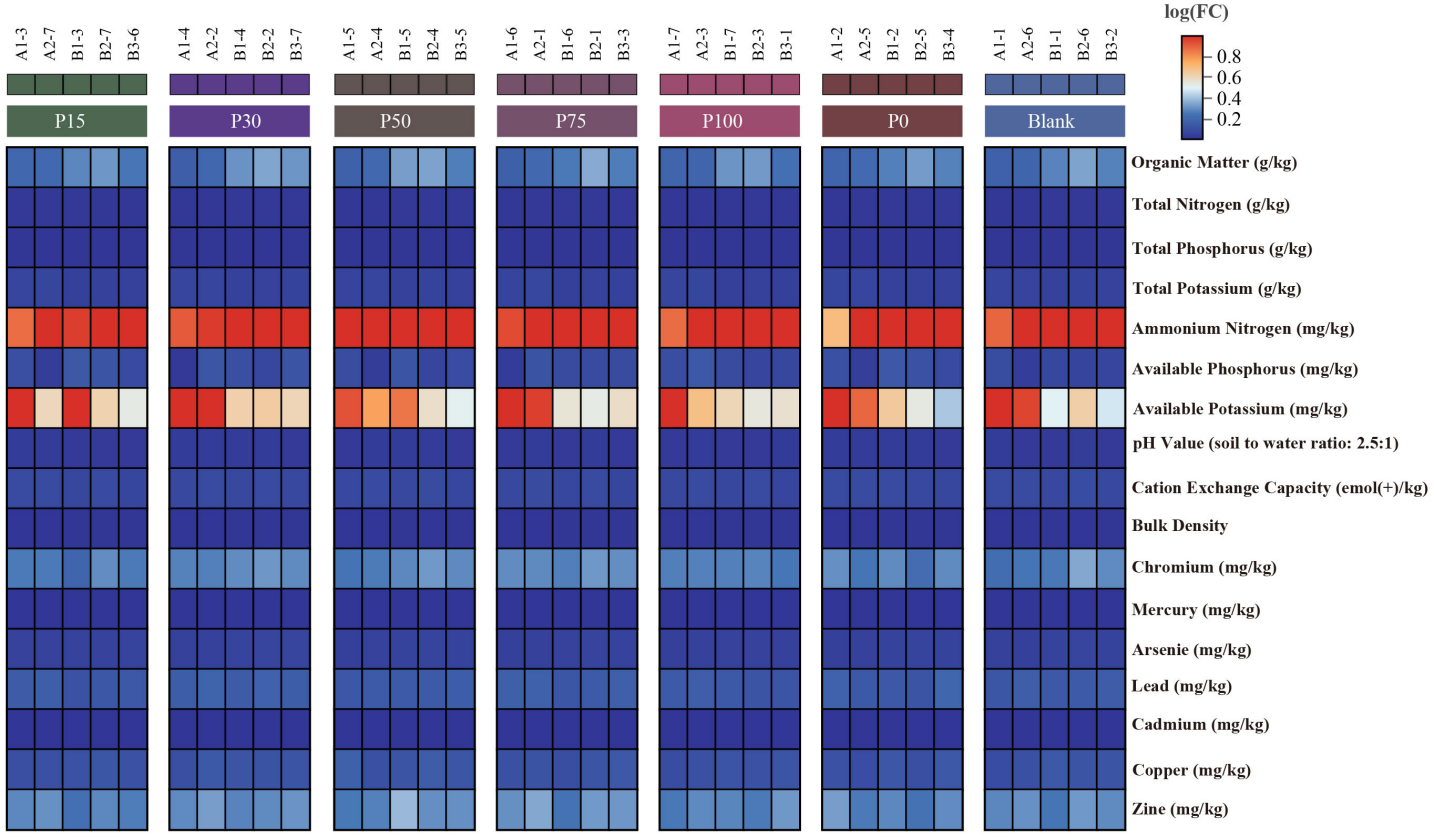
Expression of soil property indicators among different groups. Each column represents a sample, where redder colors indicate higher values and bluer colors indicate lower values.

To evaluate the effectiveness of replacing chemical fertilizers with BS, we analyzed changes in soil chemical and physical properties to assess their significance. The data presented in the heatmap reflect variations in various soil indicators among different treatment groups. Results in Figure 1 show that across the treatment groups where chemical fertilizers were replaced with different concentrations of BS, the soil contents of total nitrogen, total phosphorus, and total potassium did not significantly change compared to the P0 group without BS addition. This indicates that BS has a similar effect to traditional chemical fertilizers in enhancing soil organic matter content. The most significant difference among the groups was observed in available potassium. Aside from this, other indicators showed little variation among the groups. Overall, BS did not exhibit significant differences in its effect as a replacement for chemical fertilizers, suggesting that it can serve as a potential substitute. However, in promoting its use as a sustainable agricultural practice, long-term experiments and optimization of its replacement ratios are still required to maximize its benefits.

### 3.2 Diversity and composition of soil microbial communities

**Figure 2:**
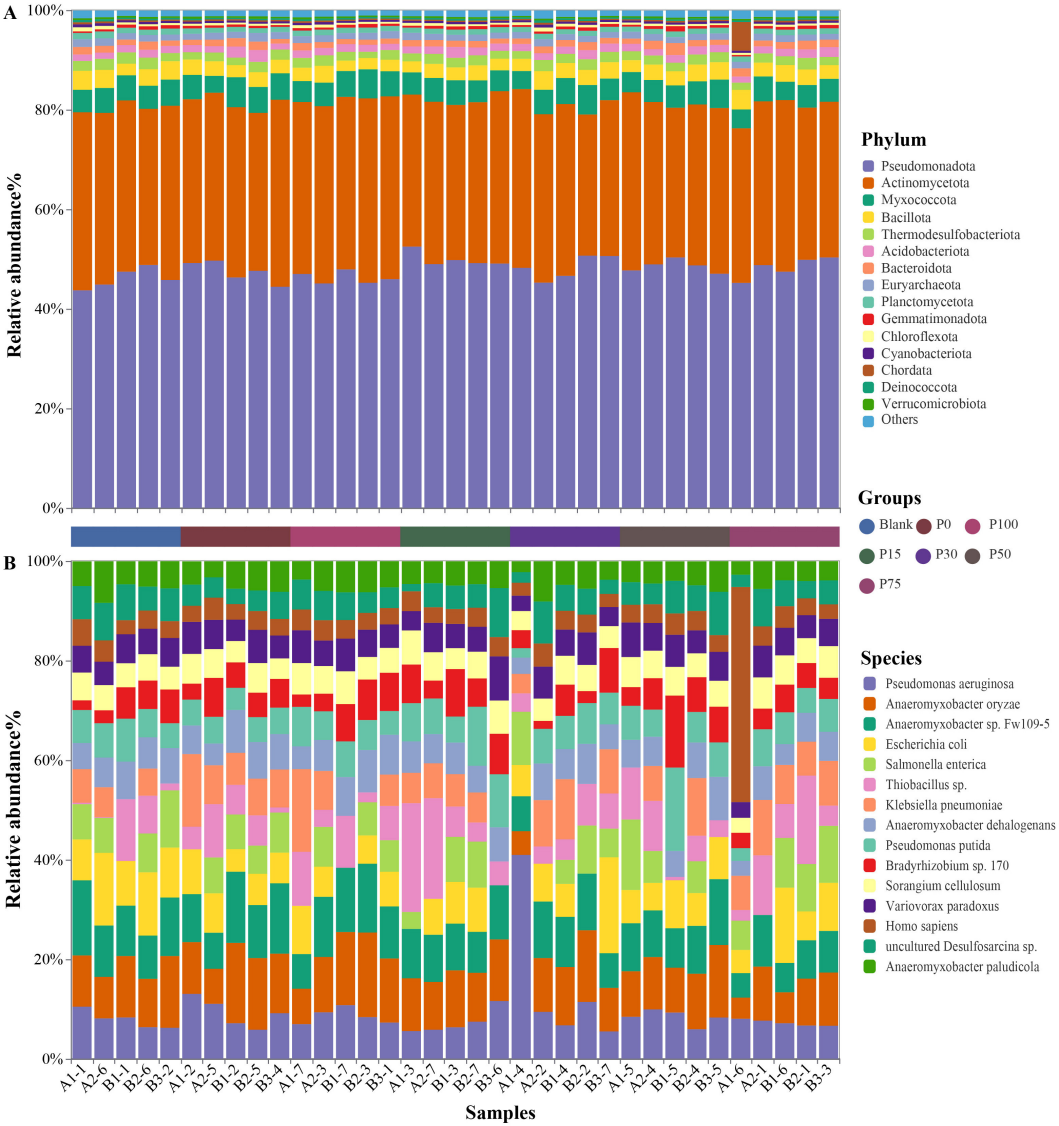
Distribution of microbial communities. **(A)** Shows the distribution of microbial communities at the phylum level among different groups; **(B)** shows the distribution of microbial communities at the species level among different groups.

After annotation, a total of 69 phyla were identified, and the soil microbial community compositions of the seven oilseed rape soil groups were relatively similar. As shown in Figure 2A, we presented the top 15 phyla based on abundance, which are *Pseudomonadota, Actinomycetota, Myxococcota, Bacillota, Thermodesulfobacteriota, Acidobacteriota, Bacteroidota, Euryarchaeota, Planctomycetota, Gemmatimonadota, Chloroflexota, Cyanobacteriota, Chordata, Deinococcota*, and *Verrucomicrobiota*. Among these, the phyla *Pseudomonadota* and *Actinomycetota* were the most dominant, accounting for approximately 80% of the total sequences in each sample, while the other 67 phyla, including *Myxococcota* and *Bacillota*, accounted for only about 20% of the total sequences.

Further investigation into the distribution characteristics of soil bacterial communities at the species level revealed that most species belonged to the phyla *Pseudomonadota* and *Myxococcota*, while the previously abundant *Actinomycetota* decreased in abundance compared to the phylum level analysis. As shown in Figure 2B, we presented the top 15 species based on abundance, which are, in order: *Pseudomonas aeruginosa, Anaeromyxobacter oryzae, Anaeromyxobacter* sp. *Fw109-5, Escherichia coli, Salmonella enterica, Thiobacillus* sp., *Klebsiella pneumoniae, Anaeromyxobacter dehalogenans, Pseudomonas putida, Bradyrhizobium* sp. *170, Sorangium cellulosum, Variovorax paradoxus, Homo sapiens, uncultured Desulfosarcina* sp., and *Anaeromyxobacter paludicola*. Some of these species have not yet been classified in the NCBI bacterial database.

**Figure 3:**
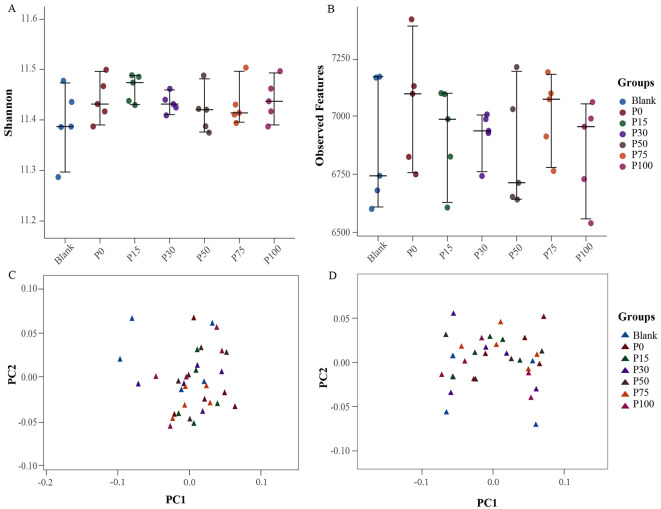
Distribution of bacterial diversity indices among different groups. **(A, B)** Represent the Shannon and observed feature indices of α-diversity, respectively; **(C, D)** represent the Bray-Curtis and Jaccard indices of β-diversity, respectively.

In terms of α-diversity, the Shannon diversity index showed no significant differences among the groups, with indices for all groups ranging from 11.2 to 11.6, indicating minimal differences (Figure 3A). This suggests that replacing chemical fertilizers with BS has little significant impact on microbial community diversity. Furthermore, the number of observed features showed a similar trend (Figure 3B). Although the P100 group exhibited a slight decrease in feature numbers, overall, the changes from the control group to the various BS replacement groups were not significant. Feature numbers remained approximately between 6,500 and 7,250, further confirming the stability of microbial diversity. In β-diversity analysis, PCA based on Bray-Curtis (Figure 3C) and Jaccard (Figure 3D) indices revealed differences in microbial community structures among the groups. In Figures 3C, D, although there was a slight clustering trend among the groups, there was no clear separation of samples from different groups. This indicates a high similarity in microbial community composition among the treatment groups, suggesting that replacing chemical fertilizers with BS did not cause significant changes in community structure.

Overall, the results of both α-diversity and β-diversity analyses indicate that replacing chemical fertilizers with BS did not have a significant impact on the diversity and composition of soil microbial communities. This finding suggests that under the current research conditions, BS can replace chemical fertilizers without altering the soil microbial structure, thereby serving as an economical and environmentally friendly fertilization scheme. Future studies can further explore the effects of BS under different environmental conditions to comprehensively evaluate its ecological benefits.

**Figure 4:**
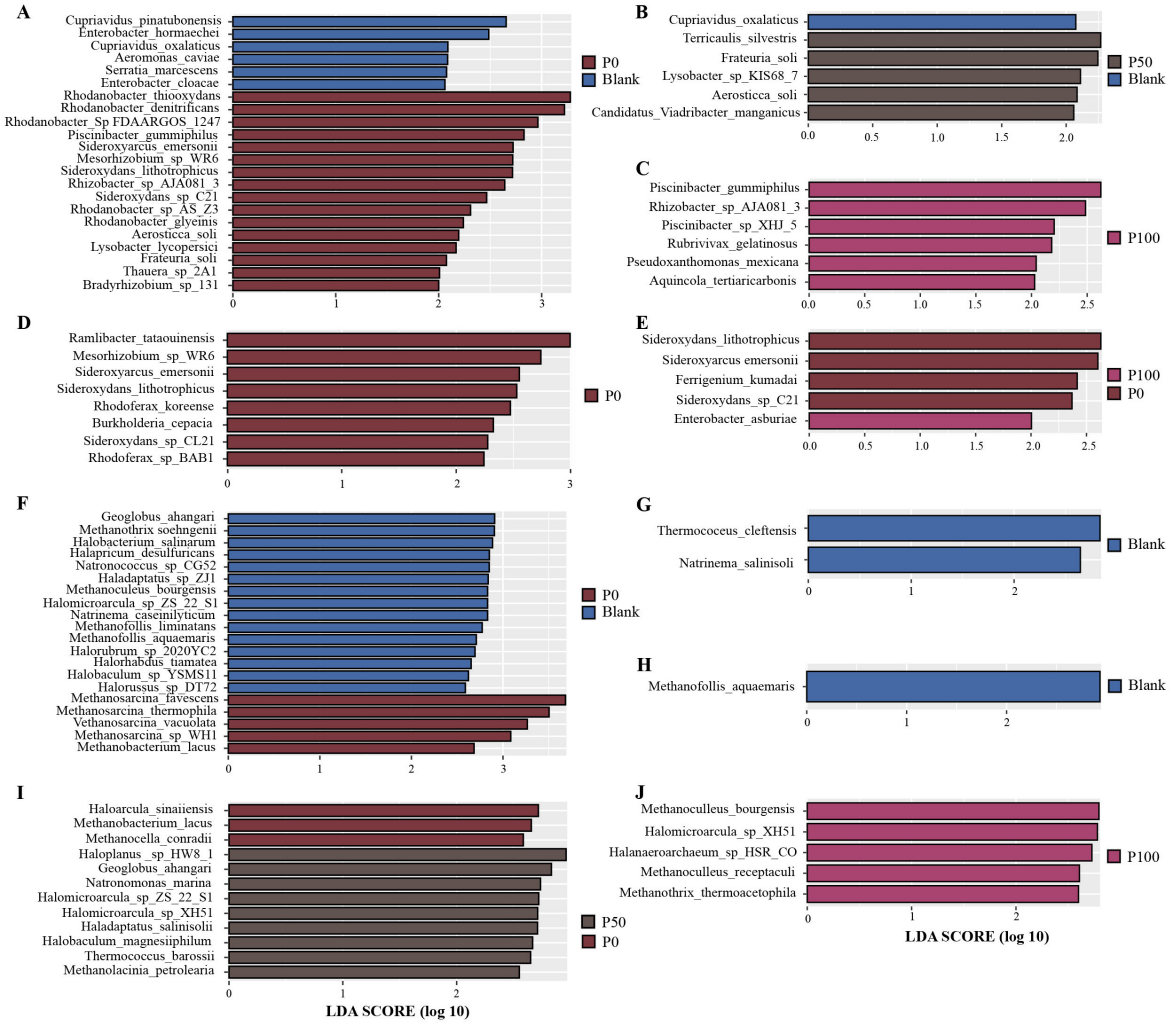
Biomarker distribution among different groups. **(A–E)** Represent bacterial biomarkers, while **(F–J)** represent archaeal biomarkers; **(A–C)** show the biomarker results between Blank and P0/P50/P100, respectively; **(D, E)** show the biomarker results between P0 and P50/P100, respectively; **(F–H)** show the biomarker results between Blank and P0/P50/P100, respectively; **(I, J)** show the biomarker results between P0 and P50/P100, respectively.

To reveal differences in microbial communities among treatment groups with different concentrations and to identify statistically significant biomarkers, we employed the LEfSe method to identify potential biomarkers. Through LEfSe analysis (LDA score ≥ 2), we were able to more precisely uncover differences in microbial communities among different treatment groups and determine significantly enriched biomarkers. As shown in Figure 4, the analysis revealed specific bacterial and archaeal biomarkers.

In the biomarker analysis of bacterial communities (Figures 4A–E), although treatments with different replacement ratios of BS caused some changes in microbial communities, there were no significant overall differences. Regarding bacteria, Figure 4A shows the differences between the control group and the P0 group; biomarkers identified in the Blank group included bacterial taxa such as Cupriavidus pinatubonensis and Enterobacter hormaechei, while the P0 group included some species of the genus *Rhodanobacter* (e.g., *Rhodanobacter thiooxydans, Rhodanobacter* sp. *FDAARGOS 1247*, and *Rhodanobacter denitrificans*). Previous studies have indicated that some species of *Rhodanobacter* possess sulfur redox capabilities, with *Rhodanobacter thiooxydans* participating in sulfur metabolism through redox reactions under anaerobic conditions (54, 55). This suggests that traditional chemical fertilizer treatments may influence the enrichment of specific sulfur-oxidizing bacteria, thereby altering the soil microbial community. As the replacement ratio of BS increased, the biomarker microbial taxa identified in the Blank group became almost undetectable in Figures 4B, C. Figures 4D, E show the differences in bacterial communities between the P0 group and the P50 and P100 groups. *Sideroxydans lithotrophicus, Sideroxyarcus emersonii, and Sideroxydans* sp. *CL21*, which were in the P0 group, were considered significantly enriched biomarkers in the P50 and P100 groups, while Enterobacter asburiae was detected as a biomarker in the P100 group. This indicates that certain bacteria that may have ecological advantages under traditional chemical fertilizer treatments become less dominant under higher proportions of BS replacement. Overall, although BS gradually replaced chemical fertilizers, it did not lead to significant changes in bacterial community structure. This suggests that BS can serve as an effective alternative to chemical fertilizers, combining economic efficiency and environmental friendliness.

In the archaeal communities, Figure 4F shows archaeal species enriched in the P0 group, such as *Methanosarcina flavescens, Methanosarcina thermophila*, and *Methanosarcina vacuolata*, while the Blank group was enriched with archaea including *Geoglobus ahangari* and *Methanothrix soehngenii*. Clearly, conventional chemical fertilizer treatments did not have a particularly significant impact on archaeal community structure. Figures 4G, H further demonstrate the differences in archaeal communities between the Blank group and the P50 and P100 groups, particularly in the Blank group where *Methanofollis aquamaris* and *Thermococcus cleftensis* were significantly enriched, and no biomarkers were found in the P50 and P100 groups. Figures 4I, J respectively display the archaeal species enriched in the P0 group compared to the P50 and P100 groups, with biomarkers identified in the P100 group including *Methanoculleus receptaculi* and *Halomicroarcula* sp. *ISR C0*, indicating that higher proportions of BS replacing chemical fertilizers may confer certain advantages for archaeal proliferation.

The impact of replacing chemical fertilizers with different concentrations of BS on bacterial and archaeal community biomarkers in the soil was minimal, and the community structure did not undergo significant changes. This indicates that BS can replace traditional chemical fertilizers without significantly altering microbial communities. In bacterial communities, the fertilized groups exhibited more biomarkers compared to the Blank group, while archaeal communities also maintained good stability after BS replacement. Overall, these results suggest that BS, as an alternative to chemical fertilizers, performs well in maintaining microbial community diversity and ecological balance, demonstrating good feasibility.

### 3.3 Associations among functional genes in soil

#### 3.3.1 Variations in the composition of ARGs

**Figure 5:**
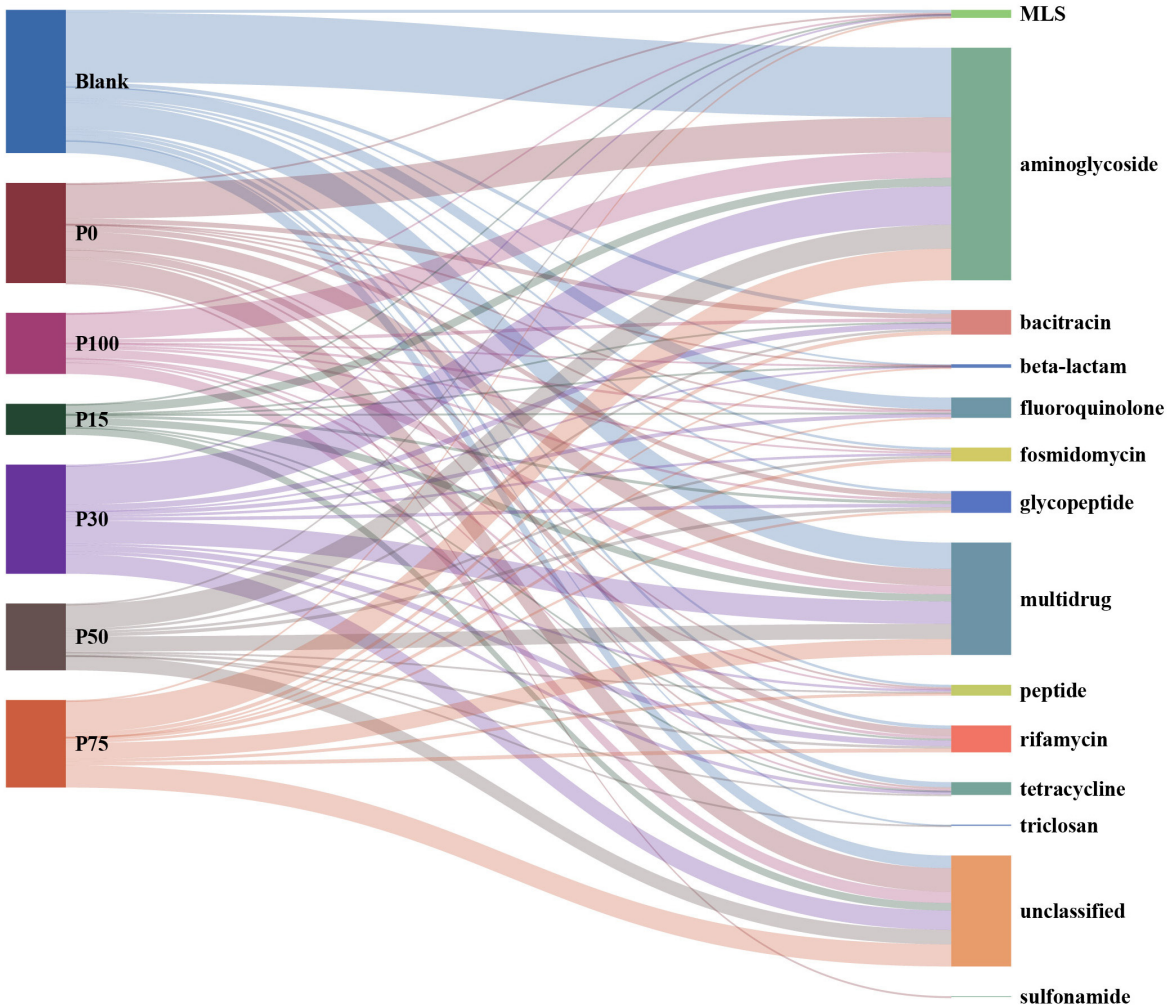
Distribution of the relationship between different concentration substitution groups and antibiotic resistance gene categories.

Antibiotics are considered one of the major breakthroughs of 20^th^-century modern medicine, playing a crucial role in controlling infectious diseases in humans and animals (56). They are commonly used to promote livestock growth and prevent infections; however, this practice has led to the accumulation of antibiotic residues and antibiotic-resistant bacteria in animal guts. These residues and resistant bacteria are ultimately released into the environment through animal feces (57). The widespread use of antibiotics poses a severe threat to global public health. To address this issue, governments have implemented measures to ban certain growth-promoting antibiotics and encourage farmers to adopt greener and more sustainable alternatives. In this study, we used BS in varying proportions to replace chemical fertilizers. By comparing our data against ARGs databases, the results are shown in Figure 5, which illustrates the relationships between different treatment groups and various categories of ARGs. A total of 1,274 sequences were successfully annotated to the database. The most annotated ARG type was aminoglycoside, accounting for approximately 39% of the total, followed by the multidrug type at about 19%. Additionally, the unclassified type also accounted for 19%; these three types constituted approximately 76% of the total. Among the 495 sequences classified as aminoglycoside, the most abundant was KDPE, with 401 sequences, accounting for 81%. Overall, the number and types of resistance genes encoded by different groups showed significant differences, especially exhibiting clear trends in specific categories of resistance genes. Aminoglycoside and multidrug were the categories with the highest numbers of encoded resistance genes across multiple groups, while the unclassified category also held a considerable proportion in each group. In the Blank group, the number of aminoglycoside category encodings was 148, significantly higher than other categories; the multidrug category had 56 encodings. Additionally, fluoroquinolone (25 encodings) and unclassified (27 encodings) also represented a large proportion. Other resistance gene categories, such as bacitracin (8 encodings) and rifamycin (7 encodings), had smaller numbers. The P0 group similarly showed prominent performance in the aminoglycoside category, with 74 encodings; the multidrug category had 36 encodings, and the unclassified category had 50 encodings. The rifamycin category had 15 encodings in this group, while the bacitracin category had 10. In the P100 group, the aminoglycoside category had 55 encodings; the multidrug and unclassified categories had 18 and 24 encodings, respectively. The P15 group showed relatively lower counts in the aminoglycoside category, with 18 encodings, but the unclassified and multidrug categories still had significant numbers, 16 and 15 respectively. The P30 group had the highest number of aminoglycoside encodings, reaching 82; the multidrug category had 48, and the unclassified category had 41. This group also showed more distribution in the rifamycin (12 encodings) and bacitracin (12 encodings) categories. The performances of the P50 and P75 groups were relatively similar; the aminoglycoside category had 51 and 67 encodings, respectively; multidrug had 32 and 34 encodings, and the unclassified category also held important proportions in these two groups, being 31 and 47, respectively.

In summary, different groups generally exhibited higher numbers of encodings in the aminoglycoside and multidrug categories, especially in the Blank, P0, and P30 groups, where these categories dominated the distribution of resistance genes, while the high-concentration groups performed less prominently. Meanwhile, the significant presence of the unclassified category in each group suggests the existence of more resistance genes that have not been fully classified. These data provide an important basis for deeper research and classification of resistance genes.

#### 3.3.2 Variations in the composition of MGEs

**Figure 6:**
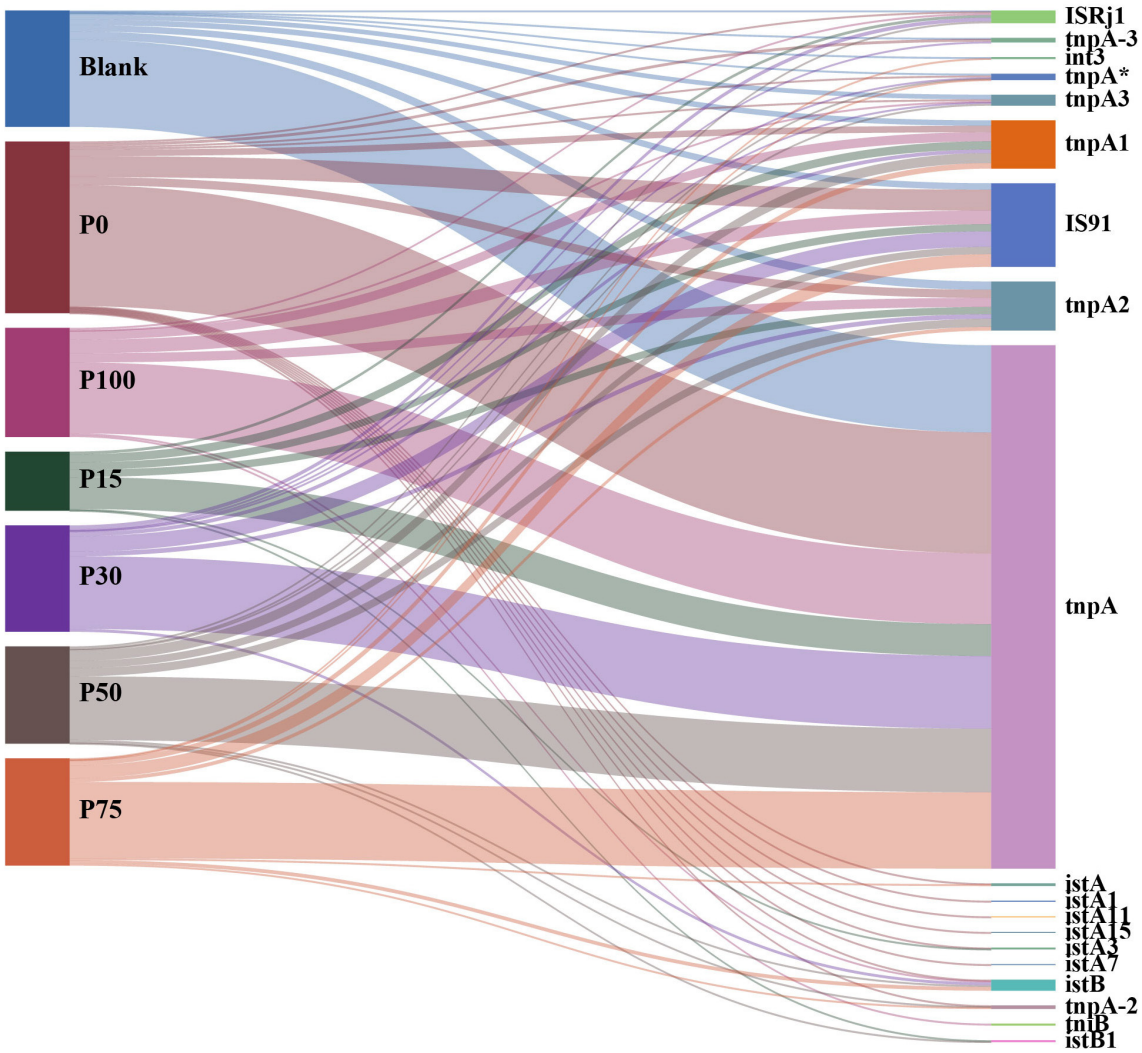
Distribution of the relationship between different concentration substitution groups and mobile genetic element categories.

Figure 6 illustrates the associations between different BS replacement concentrations and various categories of MGEs. In all replacement concentration groups, the tnpA category was significantly abundant, dominating almost all treatment groups. The widespread presence of these transposase-related MGEs may be related to their natural abundance in soil microbial communities, and this dominant distribution was not affected by the replacement of chemical fertilizers with BS, reflecting their stability in the soil environment. Additionally, other MGE categories, such as IS91, tnpA3, and int3, also maintained certain abundances across groups but did not show significant intergroup differences. It is worth noting that although some low-abundance MGE categories (e.g., istA, istB, and tnpA-2) showed slight distribution differences among different treatment groups, these changes were not significant enough to indicate that the BS replacement concentration had a significant impact on their abundance and distribution. Whether in the P100 group, which completely replaced chemical fertilizers, or in partial replacements, no significant distribution differences were observed, indicating that the community structure of MGEs has strong resilience and adaptability to the treatment of replacing chemical fertilizers with BS. Based on the results shown in the figure, we can conclude that the treatments of replacing chemical fertilizers with different concentrations of BS had minimal impact on the distribution of soil MGEs and did not lead to significant changes in MGE categories. This suggests that, at the current experimental concentrations, replacing chemical fertilizers with BS did not impose strong ecological selection pressure on mobile genetic elements in soil microbial communities, and the community structure of MGEs in soil remained relatively stable under the background of BS replacement.

#### 3.3.3 Variations in the composition of MRGs

**Figure 7:**
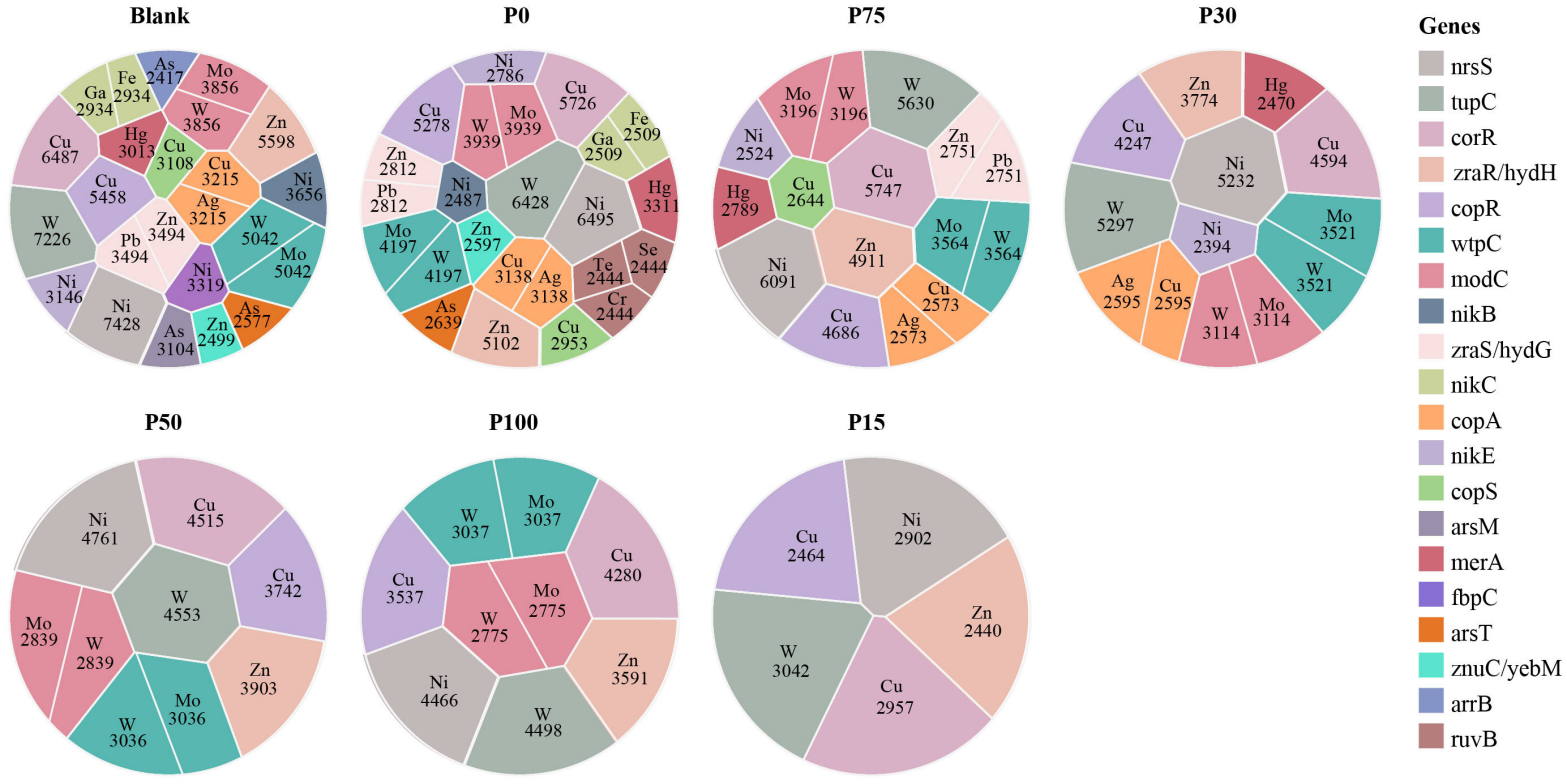
Distribution of the relationship between different concentration substitution groups and metal resistance gene categories. Each spherical symbol represents combinations of different metals and gene abundances under specific group conditions. Different colors indicate specific genes, and the labeled areas represent metal types and their relative abundance under those conditions.

We identified and analyzed MRGs in different BS replacement groups using the BacMet2 database. A total of 23 different metal types were obtained. Based on their gene abundance, we selected the top 100 for graphical representation. As shown in Figure 7, the diversity of metal types from high to low was: Blank > P0 > P75 > P30 > P50 > P100 > P15. In all experimental groups, the abundances of Cu, Ni, and W consistently dominated. These metal resistance gene categories were widely present in all replacement groups and did not show significant increases or decreases with changes in replacement concentration. The figure shows that these metal resistance genes (e.g., copA, copR, nikC, and wpC) were relatively uniform in abundance across treatment groups, indicating that these genes in the soil maintained strong stability under BS replacement treatments. Furthermore, for other metal resistance gene categories (e.g., Zn, Mo, and Ag), there were no significant distribution differences among different replacement concentration groups. These metal resistance genes (e.g., zraS, arsT, and merA) had similar distributions under different treatments and did not exhibit significant enrichment, indicating that replacing chemical fertilizers with BS did not strongly affect the structure of metal resistance genes in soil microbial communities. We can even observe that even in the P100 group, which completely replaced chemical fertilizers, the distribution of metal resistance genes remained consistent with other replacement ratios (e.g., P15, P50, and P75), further confirming the limited effect of BS replacement. Interestingly, certain specific metals (e.g., Hg and Se) appeared in some treatment groups, but their abundances were low and distributions relatively random, without significant changes with replacement ratios. We speculate that the presence of these low-abundance metal resistance genes may be more related to the background environment of the soil rather than the direct influence of BS replacement treatments.

In summary, this study indicates that replacing chemical fertilizers with different concentrations of BS did not significantly affect the structure and abundance of soil metal resistance genes. The community structure of metal resistance genes in soil remained relatively stable under different replacement conditions, suggesting that these genes may have strong adaptability to BS replacement, or that the treatment of replacing chemical fertilizers with BS was insufficient to cause significant expression differences in metal resistance genes.

#### 3.3.4 Composition of ARGs/MGEs/MRGs

**Figure 8:**
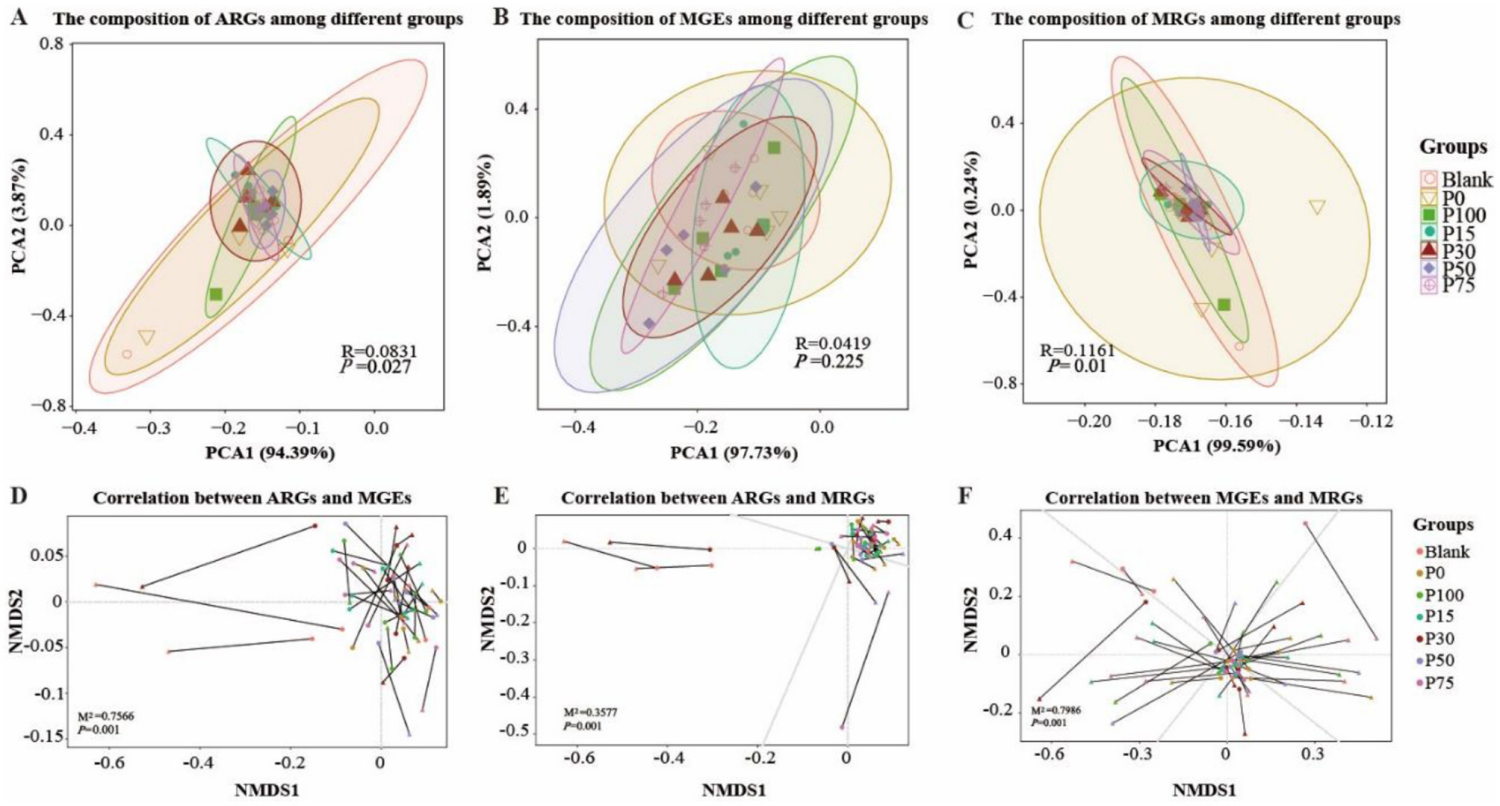
Analysis of composition and correlations among ARGs, MGEs, and MRGEs across treatment groups. **(A–C)** Principal Coordinate Analysis (PCoA) of the composition of Antibiotic Resistance Genes [ARGs, **(A)**], Mobile Genetic Elements [MGEs, **(B)**], and Multi-Drug Resistance Gene Elements [MRGEs, **(C)**] across treatment groups. **(D–F)** Non-metric Multidimensional Scaling (NMDS) analysis of the correlations between ARGs and MGEs **(D)**, ARGs and MRGEs **(E)**, and MGEs and MRGEs **(F)**, and triangles and circles denote distinct gene annotation approaches as presented in the successive titles of the figure parts.

Finally, in exploring the effectiveness of replacing chemical fertilizers with BS, we examined the differences in ARGs, MGEs, and MRGs among the groups from the perspective of gene abundance. By analyzing the changes of these genes in different treatment groups, we can evaluate the actual impact of replacing chemical fertilizers with BS. The analysis results are shown in Figure 8. In Figure 8A, the PCA analysis of ARGs shows that although the Blank and P0 groups are somewhat separated from other groups in the plot, overall, there is considerable overlap among the groups, especially from the P0 to P100 BS treatment groups. This indicates that even when completely replacing chemical fertilizers with BS, its impact on ARGs is not significantly different from other lower replacement ratios. In the PCA of MGEs, the groups similarly show large overlapping areas. Although the P100 group is more prominently separated in space, the R-value is low (0.0419) and the *P*-value is high (0.225), indicating that this separation is not statistically significant. The distribution of MRGs is similar to that of ARGs and MGEs. Although PCA1 accounts for a high percentage (99.59%), indicating that this direction is the one with the greatest variation in the data and explains most of the information, the overlap between groups is still evident, particularly in groups with lower BS replacement ratios.

Subsequently, we used non-metric multidimensional scaling (NMDS) for dimensionality reduction to compare pairwise correlations. The correlation between ARGs and MGEs (Figure 8D) shows the relationship between them. The lines in the figure represent the strength and direction of correlations between different ARGs and MGEs. In the Blank and P15 groups, a few samples show deviations and are farther apart, indicating larger differences between them. The majority are clustered together, indicating a strong correlation between ARGs and MGEs. In Figure 8E, most are clustered together, indicating a very strong correlation between ARGs and MRGs. In Figure 8F, the lines between MGEs and MRGs are longer, indicating lower correlation. The pairwise correlations are all statistically significant (*P* = 0.001). These differences in correlations may be related to the increased proportion of BS replacing chemical fertilizers, but the actual differences in gene abundance are not obvious.

Although theoretically, replacing chemical fertilizers with BS can reduce the environmental burden caused by fertilizer use, in this study, completely or partially re-placing chemical fertilizers with BS did not significantly affect the gene abundance of ARGs, MGEs, and MRGs in soil. This further illustrates that BS can be a preferred choice to replace chemical fertilizers. However, under our experimental conditions, the effectiveness of replacing chemical fertilizers with BS is limited, and further exploration is needed to demonstrate its value in practical applications.

## 4 Discussion

### 4.1 Impact of biogas slurry as a fertilizer substitute on soil properties

Biogas slurry, a byproduct of the anaerobic digestion of animal manure and crop residues, plays a significant role in enhancing the resource utilization efficiency of animal waste (58, 59). Moreover, the use of BS can effectively reduce the negative environmental impacts of animal excreta (60–62). Soil fertility is an important indicator of plant production capacity, and reasonable fertilization is one of the key means to enhance soil fertility and promote crop growth. Fertilization can replenish nutrients in the soil, improve its physical and chemical properties, and thereby effectively enhance the soil's ability to support plant growth. In this study, we conducted a 1-year field trial, and the results indicated that the substitution of chemical fertilizers with BS at different concentrations had a limited impact on the total nitrogen, phosphorus, and potassium content in the soil, with no significant changes observed across the treatment groups in these key nutrient indicators. This is consistent with previous studies, indicating that BS does not significantly increase the main nutrient content of the soil in the short term (63). Although some studies have suggested that BS can increase soil organic matter content and improve soil structure (20). In this study, the change in soil organic matter content under BS substitution treatment was not significant, possibly due to the adaptability of soil microbial communities or the need for a longer time for the decomposition of BS to fully release organic matter. Among all soil property indicators, the change in available potassium was the most significant. Particularly, the P30 and P100 groups showed an increase in available potassium, which may be due to the relatively high potassium content in BS and its easy release, thereby increasing the content of available potassium in the short term after application. Interestingly, the substitution of chemical fertilizers with BS at different concentrations did not significantly affect the content of heavy metal elements in the soil, such as chromium, mercury, arsenic, lead, cadmium, copper, and zinc, indicating that the substitution of BS does not increase the risk of soil heavy metal accumulation under current conditions. Although the effect of BS as a substitute for chemical fertilizers did not significantly surpass conventional fertilizers, it provides a more environmentally friendly fertilization option, reduces the use of chemical fertilizers, and promotes the resource utilization of organic waste, thereby helping to achieve sustainable agricultural goals.

### 4.2 Impact of biogas slurry as a fertilizer substitute on soil bacterial communities

This study shows that the impact of BS as a substitute for chemical fertilizers on soil microbial communities is small. Analysis at the phylum level indicates that the main microbial community composition is similar across all treatment groups, with *Pseudomonadota* and *Actinomycetota* being the dominant phyla, accounting for nearly 80%. This result is roughly consistent with previous studies, which have shown that *Pseudomonadota, Acidobacteriota*, and *Actinomycetota* are usually the dominant bacterial groups in agricultural soils (64–66). *Pseudomonadota* is recognized as a group of copiotrophic microorganisms, which exhibit heightened activity in eutrophic environments (67). These microorganisms are involved not only in the symbiotic nitrogen-fixing process of leguminous plants but also play a key role in the biodegradation of polycyclic aromatic hydrocarbons (68). *Actinomycetota* decomposes various organic substances, produces bioactive metabolites, and plays a role in biological control in the soil environment (69).

At the species level, there are still only minor changes in the abundance of major bacterial species across different groups, such as *Pseudomonas aeruginosa, Anaeromyxobacter oryzae*, and *Escherichia coli*, which are relatively abundant in all treatment groups and have important functions. These bacterial species are usually related to the availability of soil nutrients and the carbon-nitrogen cycle, especially having significant ecological functions in the degradation of organic matter and anaerobic environments (70–73). This consistency at the species level is consistent with other studies that have found that the short-term impact of BS substitution on soil bacterial communities is small (74, 75). The results of α-diversity analysis show that there are no significant differences in the Shannon diversity index and the number of observed features between different groups, indicating that the substitution of BS has little impact on microbial diversity. The stability of microbial α-diversity may indicate strong community resilience, which is a critical marker of soil health. This finding suggests that BS substitution effectively maintains both microbial diversity and agronomic functionality, highlighting its potential as a sustainable soil management practice. The results of β-diversity analysis confirm this again. The distribution of Bray-Curtis and Jaccard indices in PCA analysis shows that the microbial community structures between different groups are highly overlapping, with no obvious separation trend, indicating that the substitution of BS for chemical fertilizers has not significantly changed the composition of soil microbial communities. This indicates that soil microbial communities can maintain their diversity and structural stability under different concentrations of BS.

The LEfSe analysis results revealed some differences between groups. Despite significant differences in specific bacterial species between the Blank and P0 groups, such as the enrichment of *Cupriavidus pinatubonensis* and *Enterobacter hormaechei* in the Blank group, and the enrichment of *Rhodanobacter thiooxydans* with sulfur oxidation function in the P0 group. In addition, the enrichment of *Sideroxydans lithotrophicus* in the P0 group suggests that conventional chemical fertilizers may promote the proliferation of specific iron-oxidizing and sulfur-oxidizing bacteria, while no significant enrichment was observed under high concentrations of BS substitution, indicating that BS may have an inhibitory effect on these bacteria.

Although theoretical considerations suggest that the substitution of BS for chemical fertilizers could influence soil microbial diversity, the results of this study indicate that BS application has no statistically significant effect on the abundance of major soil microbial phyla or their community structure across different application ratios. To more accurately assess the potential environmental and ecological impacts of BS, it is necessary to conduct long-term and large-scale studies under diverse environmental conditions to comprehensively evaluate its effects on microbial diversity. Furthermore, the observed differences in microbial communities may be closely linked to environmental changes induced by the application of chemical fertilizers, highlighting the importance of understanding both direct and indirect effects of soil amendments.

### 4.3 Impact of biogas slurry as a fertilizer substitute on soil genetic functions

By analyzing the impact of BS at different concentrations as a substitute for chemical fertilizers on ARGs, MGEs, and MRGs in the soil, it was found that the substitution of BS had a small impact on the abundance and distribution of these genes. This is consistent with previous research findings, which have demonstrated that the judicious application of agricultural organic wastes, such as biogas slurry and biogas residue, as fertilizers can significantly reduce the abundance of antibiotic resistance genes (ARGs), particularly those associated with metal resistance (76). PCA analysis showed that although there was a certain separation between the Blank group and the P0 group and other groups in the distribution of ARGs, MGEs, and MRGs, the distribution overlap between the BS treatment groups from P0 to P100 was significant, indicating that even if chemical fertilizers were completely replaced, the impact of BS on these genes was not significant. This stability may once again highlight the adaptive capacity of soil microbial communities under different concentrations of BS. The high correlation between ARGs and MGEs (Figure 8D), Mantel R = 0.7586, *p* = 0.001, indicates the close connection between ARGs and MGEs in the horizontal gene transfer of genes, such as ARGs like tetA and intI1 may be easily spread under the synergistic action of MGEs. The high correlation between ARGs and MRGs may be the result of the co-enrichment of resistance genes and metal resistance genes in the soil. In addition, the low correlation between MGEs and MRGs (R = 0.3966, *p* = 0.001) suggests that the distribution of metal resistance genes is more influenced by the soil metal background rather than being driven by gene transfer. Overall, this study shows that the substitution of BS for chemical fertilizers has a limited impact on the abundance and distribution of soil ARGs, MGEs, and MRGs. The substitution of BS for chemical fertilizers has a small impact on the spread risk of ARGs and MRGs in the short term. Short-term studies primarily capture immediate microbial responses (e.g., transient shifts in community abundance), whereas long-term application effects necessitate attention to the rate of organic matter accumulation (e.g., annual carbon sequestration differences), the adaptive evolution of microbial functional traits and the stability of soil ecological functions (e.g., sustainability of nutrient cycling efficiency). Future research should further explore its long-term application effects, especially the potential cumulative effects under different soil types and environmental conditions, to assess its ecological safety and sustainability in agriculture.

## 5 Conclusions

Based on the analytical results of this study, the substitution of chemical fertilizers with BS is feasible in maintaining the structure and diversity of soil microbial communities. Our data results show that the impact of BS on soil microbes is minimal, indicating its potential as a sustainable agricultural practice. BS not only meets the basic nutrient needs of the soil but also achieves the resource utilization of agricultural waste without significantly altering the microbial community. It should be noted that the effectiveness of BS is influenced by its source, crop type, and soil type, and the appropriate addition of chemical fertilizers may further enhance its application effects.

As the importance of sustainable agriculture, soil health, and environmental protection becomes increasingly prominent, the application prospects of BS in soil improvement and productivity enhancement are broad. Its use not only aligns with the concepts of agricultural ecology and sustainable development but also provides new ideas for the relationship between soil health, agricultural production, and ecological balance. Using BS as a nutrient source helps reduce dependence on chemical fertilizers, decrease environmental pollution, promote the development of a circular economy, and achieve the multifaceted recycling of agricultural waste. Meanwhile, future research should integrate microbial community dynamics with crop yield and quality assessments to comprehensively evaluate the agronomic efficacy of biogas slurry.

## Data Availability

Raw sequencing files and associated metadata have been deposited at CNCB (China National Center for Bioinformation) repository with the following details: BioProject Accession Number: PRJCA033921. Direct URL: https://ngdc.cncb.ac.cn/bioproject/browse/PRJCA033921.
